# Multimodal assessment of the effects of radial pressure wave therapy on spastic muscles in patients with traumatic brain injury

**DOI:** 10.3389/fneur.2026.1847848

**Published:** 2026-08-12

**Authors:** Mika Kobayashi, Toshiko Iidaka, Takemi Murayama, Haruhi Inokuchi, Tomohiro Yamaki, Toru Ogata

**Affiliations:** 1 Department of Rehabilitation Medicine, Graduate School of Medicine, The University of Tokyo, Tokyo, Japan; 2 Department of Rehabilitation Medicine, The University of Tokyo Hospital, Tokyo, Japan; 3 Department of Preventive Medicine for Locomotive Organ Disorders, 22nd Century Medical & Research Center, Faculty of Medicine, University of Tokyo, Tokyo, Japan; 4 Rehabilitation Center for Traumatic Apallics Chiba, National Agency for Automotive Safety and Victims’ Aid, Chiba, Japan

**Keywords:** brain injuries, elasticity, muscle, shock wave, spasticity, rPWT

## Abstract

**Objectives:**

Radial pressure wave therapy (rPWT) has shown promise in reducing spastic symptoms; however, its application in patients with spasticity following traumatic brain injury, particularly in those with prolonged disorders of consciousness (pDoC), remains underreported. The study aimed to investigate the immediate effects of rPWT on spastic muscles in patients with TBI-associated pDoC.

**Methods:**

Ten patients with pDoC and upper limb spasticity and ten healthy controls received a single session of rPWT (2.5 Bar, 2000 shots) to the biceps brachii. Assessments were conducted at four time points: pre-intervention (Pre), immediately after intervention (Post), 2 days, and 7 days after intervention. Evaluation included the Modified Ashworth Scale (MAS), Modified Tardieu Scale (MTS), muscle hardness (via handheld hardness meter), and muscle elasticity (via ultrasound elastography). Changes over time and between-group differences were evaluated. For preliminary subgroup analysis, patients were divided into Good Responders and Poor Responders based on the change from Pre- to Post-intervention to determine factors affecting responsiveness.

**Results:**

MAS remained unchanged, while MTS (R1) scores improved immediately after rPWT in 9 of 10 patients. However, no sustained improvement was observed on Day 7. Patients in Good Responders tended to have higher baseline muscle hardness, higher muscle elasticity, and lower MTS (R1) values.

**Conclusion:**

rPWT resulted in an immediate, though transient changes in spastic muscle symptoms of patients with pDoC. Our preliminary analyses demonstrated the possibility that greater baseline muscle stiffness might influence responsiveness. These findings would be considered when designing protocols to evaluate the efficacy of rPWT for spasticity in patients with TBI-related pDoC.

## 1 Introduction

Spasticity is a movement disorder characterized by a speed-dependent increase in tonic stretch reflexes (muscle tension) accompanied by an increase in tendon reflexes (1). In addition, the chronic conditions of spastic muscles also involve secondary structural changes of the muscle and connective tissue (2). The pathological state of spastic muscles results from various neurological conditions such as stroke, spinal cord injury, cerebral palsy, and traumatic brain injury. Spasticity reportedly develops in 13–20% of TBI cases (3), significantly impeding the performance of activities of daily living (ADLs) and hindering rehabilitation training owing to involuntary movements such as clonus and co-contraction (4, 5).

Therapeutic interventions for spasticity include physical therapy, surgical treatments such as tendon lengthening, oral anti-spasticity medications, and botulinum toxin injection therapy (6, 7). Shock wave therapy has also been reported in combination with physical therapy to enhance the therapeutic effects of nonsurgical treatments for post-stroke spasticity (4, 5). This approach involves delivering shock waves to the affected area to transmit energy to the subcutaneous tissue, intending to modify the mechanical and biological conditions of the targeted muscles. Two types of extracorporeal shock wave therapies are used: focused shock wave therapy and radial pressure wave therapy (rPWT). Focused shock wave therapy transmits high energy to targeted lesions located in a deep area, while rPWT provides radiated energy from the skin surface and transmits it to a shallow area beneath the skin (8, 9). Compared with focused shock wave, rPWT is a less invasive and less painful treatment, which motivates practitioners to use rPWT for patients in vulnerable conditions, such as pediatric or conscious disorder cases (10, 11).

Among conditions after traumatic brain injuries (TBI), patients with prolonged disorder of consciousness (pDoC), have difficulty in manifesting their painful sensation; however, their spastic symptoms should be controlled for clothing and preventing skin ulcers. Although patients with pDoC due to TBI often have a long life expectancy and require low invasive therapeutic options for the spastic symptoms, there are no reports on the use of rPWT for treatment of the spastic muscles in patients with pDoC. Patients with TBI often experience multiple and diffuse lesions, resulting in clinical symptoms distinct from strokes with localized lesions. Considering the limitations in applying findings in stroke cases to patients with TBI, it is important to objectively evaluate changes in spastic muscle condition after rPWT intervention in this population. Understanding changes in muscle condition after therapeutic intervention is essential for establishing clinical evidence of its efficacy.

To capture the changes in the muscles of patients with pDoC after rPWT, evaluation measures should cover not only the spasticity symptoms arising from neurological lesions but also secondary changes, such as contractures and limited joint range of motion, which are caused by decreased muscle elasticity and increased connective tissue density (2). The Modified Ashworth Scale (MAS), a six-grade manual scale, is the standard for assessing spasticity (12). The modified Tardieu scale (MTS), which evaluates muscle response to extension velocity, is also used to quantify symptoms (13). However, as both the MAS and MTS are manual evaluations, their objectivity and reproducibility are limited (14). To compensate for these limitations, several evaluation devices have been introduced into clinical practice. Furthermore, muscle hardness (15) and muscle elasticity measurements (16–18) have recently been used in clinical fields, including sports medicine, as objective indices of muscle properties. Evaluation with multiple indices of muscle properties may provide a better understanding regarding the changes induced by the interventions.

In this exploratory study, we aimed to obtain basic knowledge of the underlying physiological characteristics relevant to rPWT application for spastic muscles of patients with pDoC due to TBI caused by traffic accidents. We examined the relationship between changes in clinical spasticity parameters and changes in muscle properties after rPWT intervention.

## 2 Materials and methods

### 2.1 Participants

Patients were selected from those admitted to the specialized rehabilitation hospital for traumatic brain injury in July 2024. A single session of rPWT was administered to 10 patients with spastic muscles in upper limb among those diagnosed with pDoC (patient group), and 10 healthy individuals with no particular medical history (control group). For the patient group, disease duration and the number of administered medication for muscle relaxants were extracted from medical record and consciousness was evaluated by Coma Recovery Scale-Revised, rating 0–23 assess auditory function, receptive and expressive language, visuoperception, communication ability, motor functions, and arousal level.

The inclusion criteria for the patient group were as follows: (1) presence of spasticity symptoms at the time of consent, (2) diagnosis of TBI-induced consciousness disorders, (3) TBI duration of more than 6 months, (4) age between 16 and 65 years at the time of informed consent, and (5) spasticity in the biceps brachii muscle graded MAS 2 or higher at screening.

The exclusion criteria were as follows: (1) presence of wounds near the muscles to be treated, (2) history of interruption of massage owing to discomfort or other reasons, and (3) planned changes in spasticity intervention during participation.

Written informed consent was obtained from each participant or, where applicable, from their legal representatives. Intervention was performed after obtaining consent.

### 2.2 Experimental procedure

The target muscle for intervention was the biceps brachii, with the most severely affected side (left or right) selected for intervention. Intervention was performed using an rPWT device (PHYSIO SHOCKMASTER, SAKAI Medical, Tokyo, Japan), delivering 2,000 shots to the muscle belly using a standard applicator at a frequency of 10 Hz and a pressure of 2.5 Bars while the patient was in a supine position, performed by a highly experienced physiatrist (MK). The equipment settings were based on previous literature, which used rPWT for the biceps brachii muscles (19). The applicator was located in a circular area with a 5 cm diameter centered at the distal two-thirds of the muscle. Muscle condition was examined at four time points: immediately before (Pre), after (Post), 2 days after (D2), and 1 week after (D7) intervention. Throughout the study period, rehabilitation was performed at the same frequency as normal treatment (physical or occupational therapy five times a week) along with joint range of motion training.

### 2.3 Evaluation

Clinical evaluation was performed by an experienced physiatrist, who measured the MAS and MTS scores for elbow extension just before rPWT intervention. The MTS assessment was performed following a standardized protocol that has been previously described, with participants positioned supine on a bed. R2 was defined as the maximum passive extension angle obtained at the slowest speed (V1), while R1 was defined as the angle at which resistance or catch was initially felt during elbow extension at the fastest possible speed (V3) (20, 21). The speed-dependent component MTS (R2-R1) was calculated for each patient.

Measurements were performed based on the following order: R2 (V1) → R1 (V3), with a 5-min rest period between each measurement condition to minimize the effects of thixotropy (22). Each measurement was performed twice, and the average value was used for analysis.

**Figure 1:**
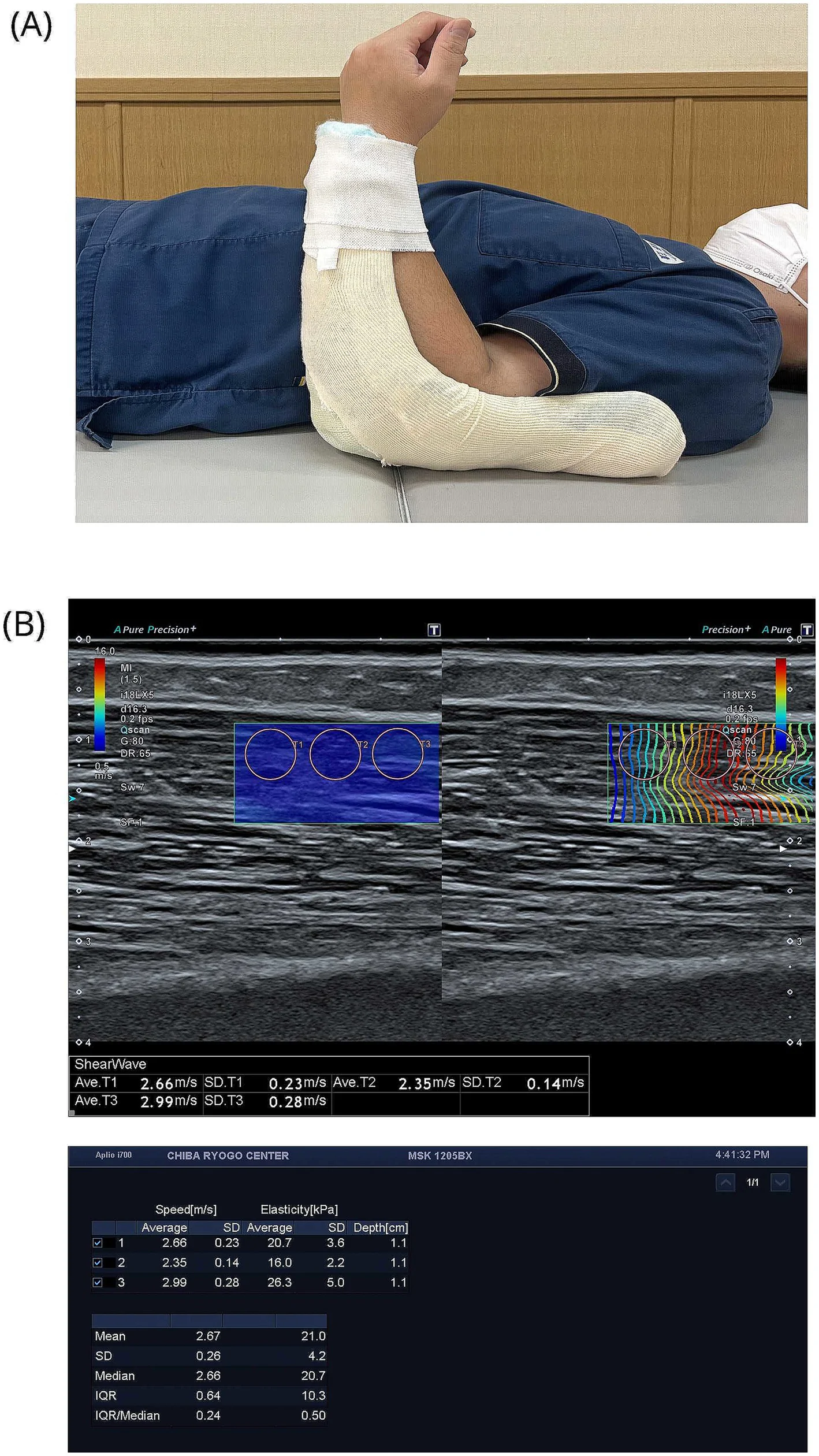
Details of the experiment. **(A)** During the experiment, the elbow was placed in a mold that could be fixed at 90° and secured with elastic bandages. **(B)** In shear-wave elastography, a circular region of interest (ROI) with a 5 mm diameter was placed on the shear-wave velocity map. Three ROIs were used to calculate muscle elasticity, and the mean value was used for analysis. After setting the ROIs, the device automatically provided both shear-wave velocity (m/s) and the corresponding elasticity values (kPa).

Muscle elasticity was assessed using ultrasound elastography (23) and muscle hardness was measured using a handheld tissue hardness meter (15) with the elbow flexed at 90°, using a water-hardening cast splint and elastic bandages (Figure 1A). To prevent voluntary contraction of the biceps brachii muscle during these measurements, surface electromyography (EMG) was employed for continuous monitoring. For ultrasonography evaluation, a mark was set at the distal two-thirds of the centerline between the elbow and acromion. This marked point was used as the center of the examination, and the presence of the biceps brachii muscle within 2 cm from the surface was confirmed using B-mode ultrasonography. To ensure the alignment of the long axis of the linear ultrasonography probe with muscle fiber orientation, the center point and probe position were marked to allow for reproducible positioning.

Muscle elasticity was assessed via ultrasound elastography (Aplio Verifier system, Canon Medical Systems, Otawara-shi, Japan) using a linear probe (i18LX5) in shear wave mode. Three 5-mm circular regions of interest were selected from the ultrasound image, and the average value was analyzed (Figure 1B).

Muscle hardness was measured using the PEK-1 device (Imoto Manufacturing Co., Ltd., Kyoto, Japan) at the same location as the ultrasound measurement center point. The device was applied five times, and the average of three measurements, excluding the maximum and minimum values, was calculated.

Furthermore, to explore the character of the responsiveness to rPWT for preliminary purpose, patients were divided into two groups—responders (Good Responders) and non-responders (Poor Responders)—based on the change in MTS (R1) from baseline to immediately after intervention. We adopted the median-based dichotomization method (24, 25) and examined factors contributing to differences in intervention effects.

### 2.4 Statistical analysis

As the number of participants was limited, the analyses were intended for deriving a preliminary character of the short-term changes after rPWT to provide basic information to future studies. Data were analyzed using JMP Pro18.1.1 (Statistical Discovery LLC, Cary, NC, USA). A Wilcoxon rank-sum test was used to compare height and weight. Fisher’s exact test was used to examine the characteristics of patients. Pearson correlation analysis was used to analyze the relationships between the spasticity assessment items before intervention. To analyze the changes after rPWT, measurement values at each post-intervention time point were compared with baseline values within the patient group. As baseline conditions varied among patients, generalized linear mixed models (GLMMs) were used to examine changes in joint angles over time, with patient included as a random effect. Post-hoc comparisons between baseline and each post-intervention time point were adjusted using Dunnett’s correction. Changes in muscle properties over time were analyzed using one-way repeated measures ANOVA. Differences in the subgroups were examined using Student’s t-test for independent comparisons or two-way repeated measures ANOVA for factorial comparisons involving times followed by post-hoc comparison using Holm’s correction, if needed. *p*-values < 0.05 were considered statistically significant.

For MAS scoring, a value of 1.5 was assigned for a grade of 1+, and the median and interquartile range (IQR) values were calculated and analyzed Kruskal-Wallis test (26, 27).

## 3 Results

### 3.1 Participant characteristics

No significant differences were observed in sex, age, height, or weight between the patient and control groups (Table 1). The patient group had a mean ± SD disease duration of 22.2 ± 7.0 months and a mean total Coma Recovery Scale-Revised of 16.2 ± 7.9, indicating consciousness impairment. The median number of muscle-relaxing medications was 1.5 (range, 0–4) (Table 2).

**Table 1:** Characteristics of patients with disorders of consciousness and healthy controls.

Variables	Pt	Cont	*p*-value
*N*	10	10	
sex M: F	7:3	5:5	0.65
age	34.5 ± 15.2	43.9 ± 10.4	0.16
high (cm)	167.9 ± 8.0	166.5 ± 6.9	0.85
weight (kg)	56.2 ± 4.4	61.3 ± 8.5	0.09

*p*-values for age, height, and weight were calculated using the Wilcoxon rank-sum test, and sex ratio was calculated using the Fisher’s exact test.

**Table 2:** Characteristics of prolonged disorders of consciousness in the patient group.

ID	Disease period (m)	Brain injury	Main mechanism of brain injury	Main side of brain injury	CRS-R	Drug
P1	25	Rt-ASDH, Rt-Thalamus	Focal brain injury	Rt	23	Levetiracetam 1,000 mg/Lacosamide 200 mg
P2	24	Rt-ASDH, Bifrontal contusion (Rt > Lt)	Focal brain injury	Rt	23	Dantrolene sodium 150 mg/Levetiracetam 1000 mg
P3	15	Lt-ASDH, Lt-fronto-parieto-occipital contusion	Focal brain injury	Lt	12	Levetiracetam 500 mg
P4	15	Quadrigeminal cistern SAH, midbrain/corpus callosum/bilateral cortex multiple bleedings	Diffuse brain injury	Bilateral	4	Levetiracetam 1000 mg/Dantrolene sodium 100 mg
P5	29	Rt-ASDH, Rt-fronto-tempro-occipital contusion	Focal brain injury	Rt	23	Valproate sodium 800 mg
P6	16	Rt-frontal/Lt-basal ganglia multiple bleedings	Diffuse brain injury	Bilateral	23	Valproate sodium 800 mg/Tizanidine 6 mg/Dantrolene sodium 25 mg /Clonazepam 1 mg
P7	17	Lt-ASDH, Lt-basal ganglia/corpus callosum & Rt-basal ganglia/midbrain contusion	Diffuse brain injury	Lt	23	Levetiracetam 1000 mg
P8	37	Bifrontal-SAH, bifrontal contusion	Diffuse brain injury	Bilateral	19	Lacosamide 200 mg
P9	17	Lt-ASDH, Bifrontal & Lt-fronto-temporal contusion	Focal brain injury	Lt	8	(−)
P10	27	Lt-ASDH, Bifronto-tempro-parietal contusion	Focal brain injury	Bilateral	4	Levetiracetam 3,000 mg/Lacosamide 200 mg/Gabapentin 1200 mg

ASDH, acute subdural hematoma; SHA, subarachnoid hemorrhage.

### 3.2 Spasticity assessment

At baseline before rPWT, spasticity in the biceps brachii of the patient group was assessed using the MAS, yielding a median score of 2 (IQR 2–2). One participant who showed a lower MAS score than at screening but had clinically apparent spasticity was included in the study. In the MTS evaluation, MTS(R1) was −86.8 ± 20.4° (mean ± SD), MTS(R2) was −41.3 ± 24.2°, and MTS(R2-R1) was 45.5 ± 22.7°.

### 3.3 Muscle characteristics assessment

**Figure 2:**
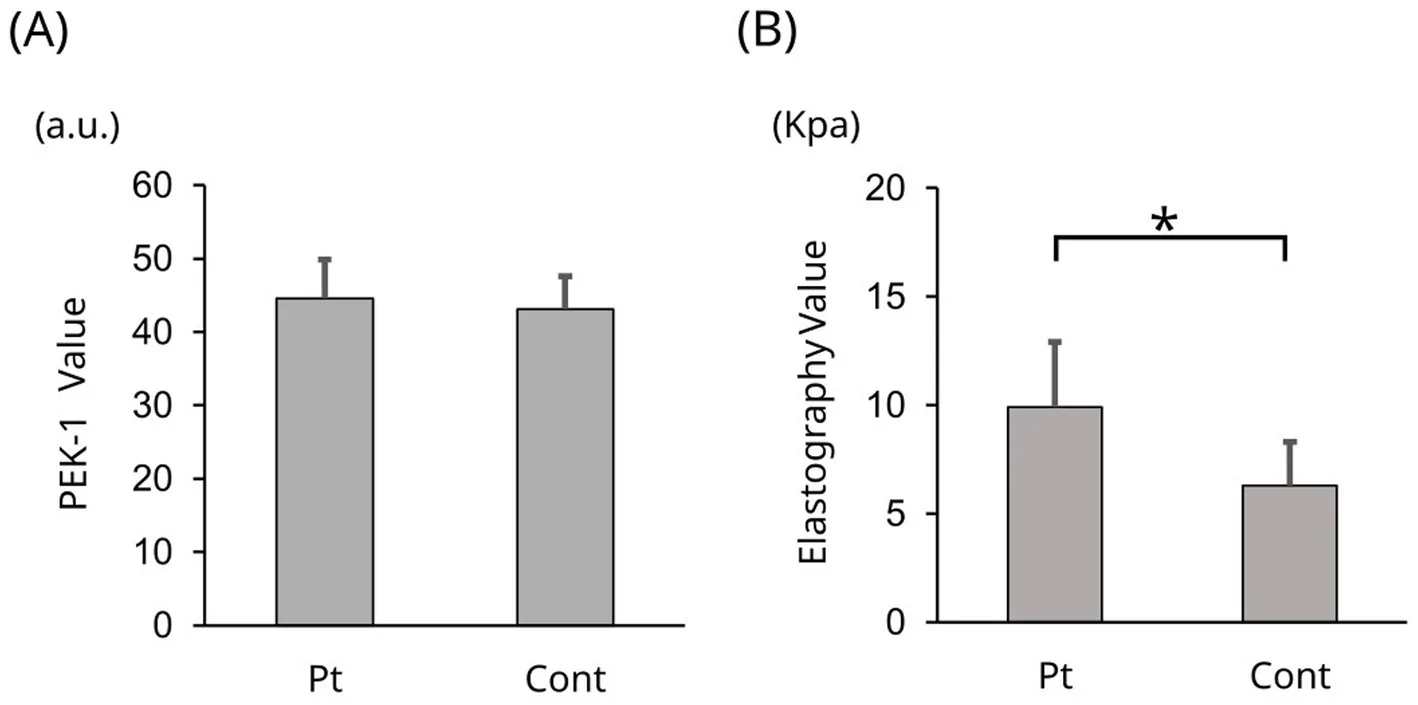
Muscle characteristics of the biceps brachii before intervention. **(A)** Muscle hardness of patients before intervention and controls (PEK-1 value). **(B)** Muscle elasticity of patients before intervention and controls (Elastography value). *p < 0.01.

Group comparison of muscle characteristics at baseline showed that muscle elasticity measured via elastography was significantly higher in the patient group than in the control group (patient group: 9.91 ± 3.03, control group: 6.32 ± 2.01, Wilcoxon, *Z* = 2.68, *p* = 0.007), although, no significant difference was observed in muscle hardness measured using the PEK-1 device (patient group: 44.6 ± 5.3, control group: 43.1 ± 4.5, *Z* = 0.6, *p* = 0.55) (Figure 2).

### 3.4 Association between spastic conditions and muscle characteristics

**Figure 3:**
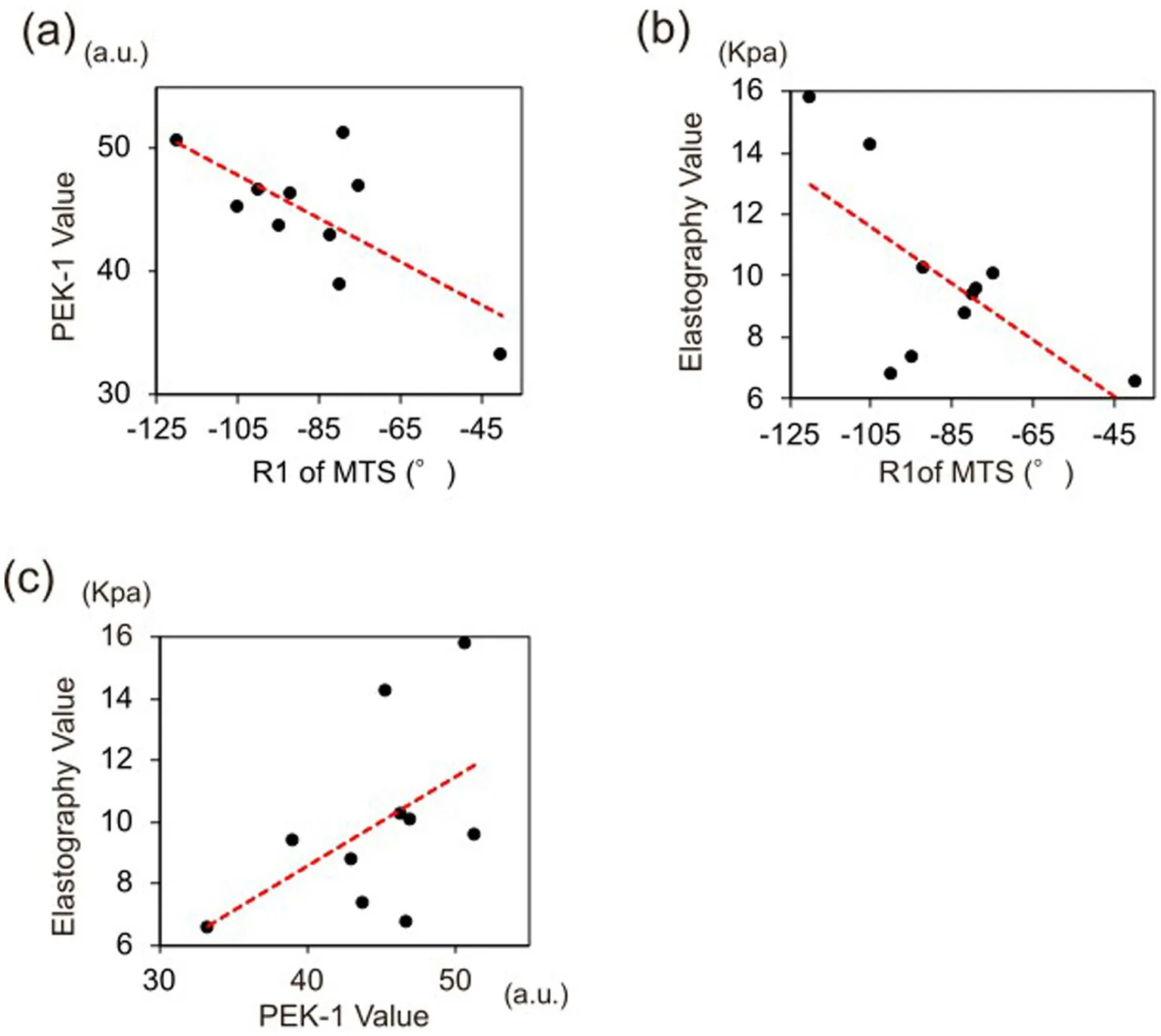
The correlation of the values of muscle characteristics. **(A)** Correlation between MTS(R1) and muscle hardness (PEK-1 value) in the pre-intervention state. **(B)** Correlation between MTS(R1) and muscle elasticity (Elastography value) in the pre-intervention state. **(C)** Correlation between muscle hardness and elasticity in the pre-intervention state. MTS, Modified Tardieu scale; MTS(R1), The angle initially felt during elbow extension at the fastest possible speed.

To investigate the relationship between baseline indicators in the groups, correlations between MTS (R1), muscle hardness, and muscle elasticity were evaluated. Significant correlations were observed between MTS (R1) and muscle hardness (PEK-1) (R = −0.71, *p* = 0.021), and between MTS(R1) and elasticity (R = −0.65, *p* = 0.043), but no correlation was observed between muscle hardness and elasticity (R = 0.52, *p* = 0.13) (Figure 3).

### 3.5 Intervention response

**Figure 4:**
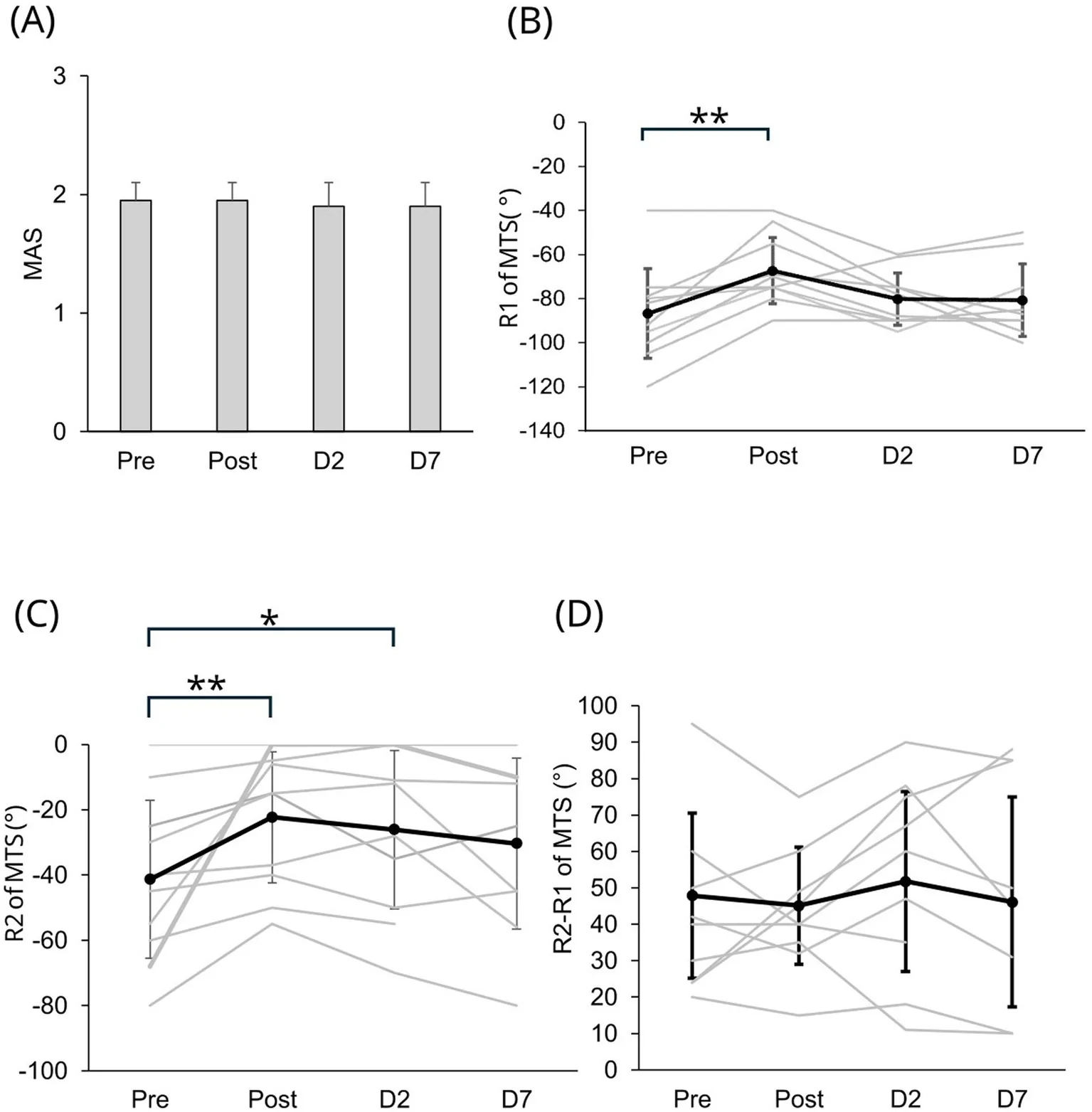
Temporal changes in MAS and MTS, manual assessment methods for spasticity. **(A)** Temporal changes in MAS. **(B)** Temporal changes in MTS(R1). **(C)** Temporal changes in MTS(R2). **(D)** Temporal changes in MTS(R2-R1). Grey lines in panels **(B–D)** represent individual changes, while dark lines represent group mean values. Data were analyzed using generalized linear mixed models. Post-hoc comparisons with baseline were adjusted using Dunnett’s correction. Pre: Before intervention, Post: Immediately after intervention. MAS, Modified Ashworth scale; MTS(R2), The maximum passive extension angle; MTS(R2-R1), The angle representing the speed-dependent component. *p < 0.05, **p < 0.01.

In the patient group, MAS scores showed no significant changes pre- and post-intervention (Figure 4A) (Kruskal–Wallis test, H = 0.62, df = 3, *p* = 0.89). Owing to large individual differences in the baseline values for MTS (R1), (R2), and (R2-R1), these variables were analyzed using the GLMM, with the measurement timing set as a fixed effect and subject as a random effect. The GLMM revealed a significant effect of time on MTS (R1) and MTS (R2). Compared with baseline (Pre) values, MTS (R1) improved at Post (*p* = 0.002), whereas MTS (R2) improved at Post (*p* = 0.009) and D2 (*p* = 0.043) timepoints. No changes were observed at D7 in either MTS (R1) or MTS (R2). No significant changes were observed in MTS (R2-R1) values at any time point (Figures 4B–D). Evaluation of muscle properties showed that neither muscle hardness (PEK-1 value) [*F*(3, 27)=0.752, *p* = 0.53] nor muscle elasticity [*F*(2.9, 26.4) = 1.541, *p* = 0.228] showed significant changes over time.

At the individual level, 9 out of 10 patients showed immediate reductions in MTS (R1) values immediately after rPWT, suggesting consistent effects. Whereas MTS (R1) values at D2 showed considerable variability.

### 3.6 Subgroup analysis of intervention response

Patients were divided into two subgroups to investigate factors influencing the response to rPWT. We divided the participants into two groups using a median split of the change in MTS (R1) values from baseline (Pre) to immediately after rPWT (Post) (range of change: 0–47°; median [IQR]: 22° [5–30°]). Participants with a change equal to or greater than the median were classified as the good responder (GR) group (*n* = 5). Meanwhile, those with a smaller change were classified as the poor responder (PR) group (*n* = 5). No significant differences were observed between the two groups in age, disease duration, or level of consciousness (Wilcoxon rank-sum test: *p* = 0.257, 0.17, and 0.21, respectively).

**Figure 5:**
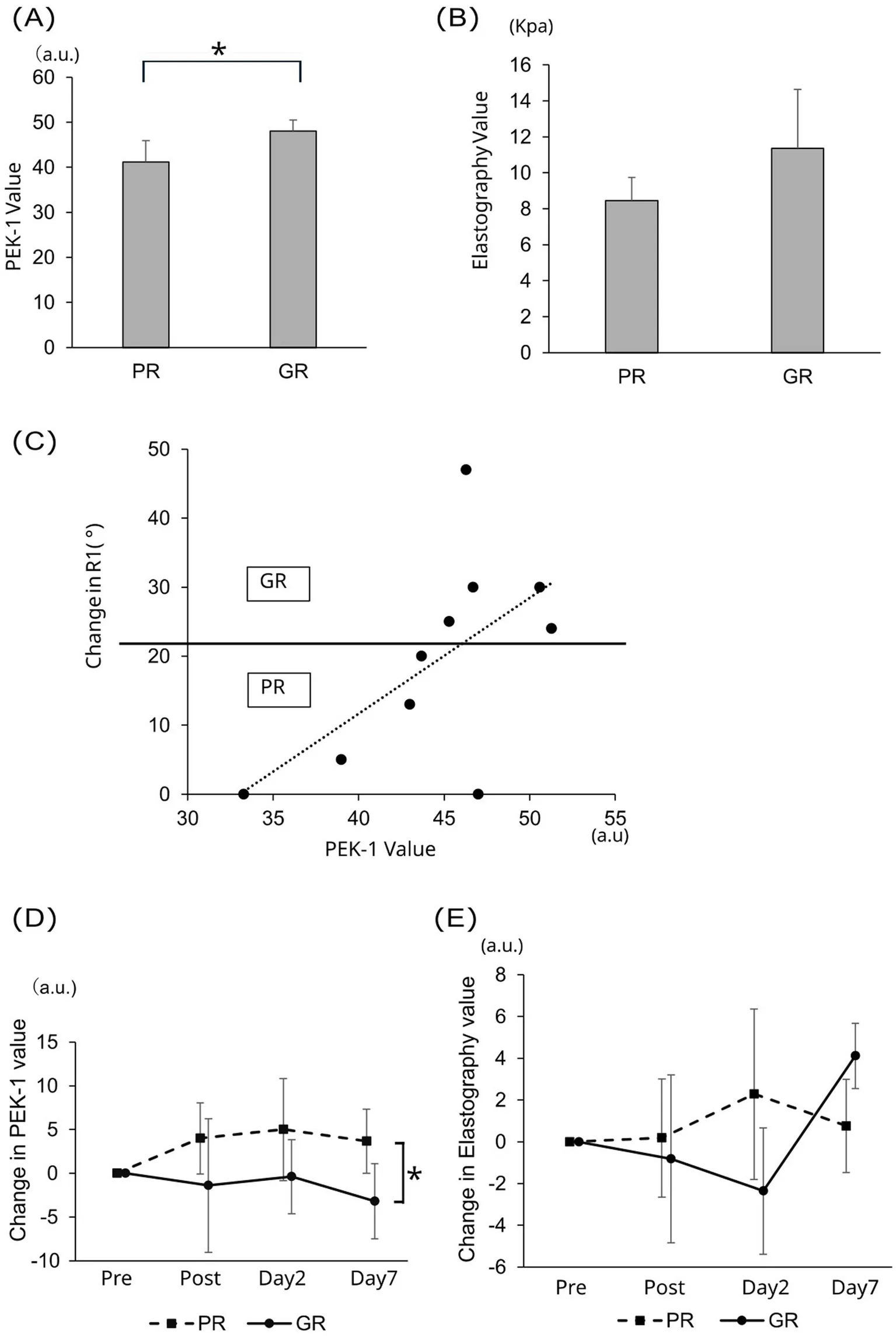
The comparison between two groups according to the response to the intervention. **(A)** Muscle hardness pre-intervention. **(B)** Muscle elasticity pre-intervention. **(C)** Scatter plot showing the relationship between pre-intervention PEK-1 values and changes in MTS(R1) from pre to post intervention in both GR and PR. The horizontal line indicates the median value used to divide the two groups. **(D)** Changes in muscle hardness from pre-intervention. **(E)** Changes in muscle elasticity from pre-intervention. Panels **(D,E)** were analyzed using two-way repeated-measures ANOVA followed by Holm-adjusted post-hoc comparisons. PR, Poor responders; GR, Good responders, *p < 0.05.

At pre-intervention, muscle hardness was significantly higher in the GR group than in the PR group (48.0 ± 2.7 vs. 41.2 ± 5.3). The mean difference between the GR and PR groups was 6.8 units (95% CI: 0.7 to 12.9, Hedges’ *g* = 1.48, *p* = 0.032). Muscle elasticity at pre-intervention also showed a higher tendency in the GR group (11.36 ± 3.65 kPa vs. 8.46 ± 1.44 kPa), but the difference was not statistically significant (mean difference = 2.90, 95% CI: −1.15 to 6.95, *p* = 0.137) (Figures 5A,B).

Conversely, MTS (R1) and MTS (R2) values were lower in the GR group than in the PR group: −99.2 ± 15.2° vs. −74.4 ± 20.6° (*p* = 0.06); −54.6 ± 26.7° vs. −28.0 ± 17.5° (*p* = 0.10), respectively, with no significance. The value of MTS (R2-R1) was 44.6 ± 32.5° vs. 46.4 ± 15.0° (*p* = 0.91). To further examine the relationship between pre-intervention muscle hardness and response to rPWT, we plotted these variables and identified a moderate correlation, which did not reach statistical significance (R = 0.60, *p* = 0.07) (Figure 5C).

Temporal changes in each group’s variables from baseline are shown in Figures 5D,E. For muscle hardness, a significant main effect of the group was observed [*F*(1, 8) = 6.1, *p* = 0.039], whereas neither the main effect of time nor the group-by-time interaction was significant. No significant difference in muscle elasticity was observed between the groups.

## 4 Discussion

In this study, rPWT induced immediate changes in upper-limb spastic muscles in patients with pDoC, with individual differences. While studies have been conducted on the effects of rPWT on upper-limb spasticity in post-stroke patients (19, 28, 29), our study is the first to examine its effects in spastic muscles in patients with pDoC as the underlying condition.

Patients with pDoC often experience communication difficulties, making subjective assessment of treatment effects challenging. Moreover, the use of systemic medications such as antispasticity drugs, may increase the risk of swallowing disorders or aspiration pneumonia, thereby limiting their use in this population. Botulinum toxin therapy also has limitations with insufficient dosage to cover all of the symptomatic limb muscles, which may hinder treatment efficacy. Considering this background, it is important to optimize the use of rPWT as a safe, local physical therapy and, as a first step, to obtain detailed information on the changes induced by this intervention.

In the present study, most patients showed immediate effects following a single application of rPWT; however, effects at 7 days after the treatment could not be confirmed through MTS evaluation. Considering the lack of change in either MAS score or MTS (R2-R1), the observed changes cannot be attributed to neural spasticity reduction. The changes may reflect shifts in passive stiffness or contracture, which also are also limited in terms of sustained influences. These results are different from previous reports (19, 28, 29), which generally involved multiple rPWT applications and obtained sustained improvement in MAS scores. Therefore, multiple applications may be required to achieve long-term symptom improvement. Furthermore, even in short-term changes, individual differences in intervention response were observed, which may be due to variations in the pathophysiology of spastic muscles. Therefore, patient characteristics were investigated to identify muscle condition factors influencing the responsiveness to rPWT. The analysis of GR and PR group data revealed that patients with more pronounced immediate effects following rPWT had higher baseline muscle hardness, muscle elasticity, and absolute the values of MTS (R1) values, indicating greater muscle stiffness as a possible factor influencing the responsiveness. Notably, the sample size of the subgroup analysis was very small; therefore, the finding should be interpreted as hypothesis-generating rather than conclusive.

In this study, we evaluated muscle properties using two methods. The first was muscle hardness (PEK-1 value), which represents the passive stiffness of superficial soft tissues, including muscle and fascia, to vertical external forces. On the other hand, muscle elasticity (elastography value) reflects the viscoelastic properties of the muscle and is measured using the shear wave velocity propagating transversely through the tissue. Both methods share commonalities; however, they reflect distinct characteristics. The fact that only muscle stiffness (PEK-1) differed between the GR and PR groups during the post-intervention period indicates that rPWT may have a more noticeable effect on the hardness of superficial soft tissue.

Increased muscle hardness and elasticity reflect the presence of severe spasticity, secondary contractures, or both (30, 31). This muscle property reportedly influences the stretch reflex through changes in tension applied to muscle spindles (32, 33). Therefore, we assumed that increased muscle hardness (PEK-1 values) provided a margin for improvement in both contracture and reflex-related factors during rPWT, leading to the observed improvement in R1. Therefore, evaluating the severity of spasticity prior to intervention with rPWT may be beneficial to achieve effective clinical outcomes. Previous reports have indicated that rPWT may act on spasticity, contracture, or both (34, 35). In the present study, immediate effects were observed in both values of MTS (R1), which indicate speed-dependent muscle responses, and values of MTS (R2), which indicate the maximum passive ROM. Given these findings, it is difficult to conclude whether rPWT affects spasticity or contracture.

In addition to manual evaluation, we evaluated muscle characteristics by assessing hardness and elasticity. Both variables were useful for capturing spastic muscle characteristics before the intervention; however, no changes in muscle elasticity were observed after rPWT. Regarding elasticity in spasticity treatment, a previous study revealed changes in elasticity between before and after botulinum toxin treatment (23). However, in that study, the period between intervention and evaluation was long, and the effects of range-of-motion training conducted in addition to botulinum toxin treatment may have affected the elasticity change, making it difficult to compare their results with our results. In contrast, muscle hardness tended to be low after intervention for 7 days in the GR group in the present study. This change in muscle hardness may reflect indirect effects of concomitant rehabilitation after rPWT. Even if the effect of rPWT remains short, the reduced hardness may interact with the following ROM exercises. Therefore, caution should be paid to the contents of rehabilitation in assessing the effects of rPWT.

The mechanism of action of rPWT still remains unclear; however, rPWT reportedly induces degeneration at the neuromuscular junctions in rats (36–38). The immediate changes observed in the MTS (R1) in the present study may be attributed to both changes in neural excitability and structural changes in muscle fibers, as supported by the observed changes in muscle stiffness. To differentiate between these two contributing factors, future research should incorporate neurophysiological evaluations in addition to clinical and muscle property evaluations. This approach will help with the selection of optimal treatments tailored to individual spasticity conditions.

This study has some limitations. First, we evaluated only the effects of a single session of rPWT to analyze its direct effects and to explore the relationship between spastic conditions and muscle properties. Evaluation of the therapeutic efficacy of rPWT was beyond the scope of this study. From the perspective of establishing therapeutic evidence, future studies should consider the number of treatment sessions required to achieve long-term effects. In addition, differences in stimulation intensity (Bar), the number of shots, and irradiation sites (the muscle belly or muscle-tendon junction) may also influence treatment efficacy (39). The current protocol of 2000 shots per muscle (2.5 Bar) is equivalent to those reported in previous literature. Many studies lack clear documentation regarding the irradiation site; however, in this study, irradiation was applied to a limited area in the central muscle belly. Identifying the optimal stimulation site remains a future task to obtain reproducible effects. The detailed information provided in this study may contribute to the design of protocols for future clinical trials.

Second, the changes in muscle properties obtained in this study are interpreted as changes in muscle structure; however, the influence of sustained muscle contraction (spastic dystonia) should also be considered. Surface electromyography was used during measurement to confirm that muscle contractions were absent; nonetheless, it is important to note that dystonic components cannot be completely excluded.

Third, the sample size was small, comprising only 20 participants (10 patients). The findings in this study should be regarded as preliminary findings which will provide basic information for future studies. In particular, the subgroup analysis may have been influenced by observational bias and should be considered with care. The study was conducted at a medical facility specializing in TBI, where a relatively homogeneous patient group was enrolled. A larger sample size may have increased the involvement of heterogeneous conditions. To examine the mechanisms underlying spasticity treatment, it is important to consider whether to prioritize subject uniformity or increase in the number of cases. Lastly, because the participants of this study were limited to cases with pDoC following TBI associated with spasticity, caution is required when generalizing the findings of this study to spasticity symptoms associated with other disease conditions.

In conclusion, the findings of this study revealed that a single session of rPWT induced immediate, though transient—lasting less than 7 days, changes in muscle spastic symptoms in patients with pDoC. Our preliminary subgroup analysis raised the possibility that the variation in responsiveness among the patients might be influenced by pre-intervention MTS (R1) values and muscle hardness. The detailed understanding of the muscle hardness in nature, together with the component affected by rPWT require further investigation. We propose a hypothesis that the pre-treatment muscle properties influence rPWT responsiveness, which would be considered for designing protocols for future studies to evaluate the efficacy of rPWT for spasticity.

## Data Availability

The raw data supporting the conclusions of this article will be made available by the authors, without undue reservation.
